# Associations of cardiorespiratory fitness with cardiovascular disease risk factors in middle-aged Chinese women: a cross-sectional study

**DOI:** 10.1186/1472-6874-14-62

**Published:** 2014-05-01

**Authors:** Wenfei Zhu, Steven P Hooker, Yuliang Sun, Minhao Xie, Hao Su, Jianmin Cao

**Affiliations:** 1 School of Nutrition and Health Promotion, Arizona State University, Phoenix, AZ 85004, USA; 2 Key Laboratory of Exercise and Health Science of the Ministry of Education, Shanghai University of Sport, Shanghai 200438, P. R. China; 3 Sport Science College, Beijing Sport University, Beijing 100084, P. R. China; 4 Sport Biochemistry Program, Sport Science College, Beijing Sport University, No.48 Xinxi Road, Beijing, Haidian District 100084, P. R. China

## Abstract

**Background:**

High levels of physical activity (PA) and cardiorespiratory fitness (CRF) are each associated with a favorable cardiovascular disease (CVD) risk profile. However, the relationship between CRF and obesity is still inconsistent across studies, and there has been no thorough exploration of the independent contribution of CRF to different CVD risk factors in Chinese women. This study investigated the relationship between CRF and CVD risk factors in 40–49 year old women in Beijing.

**Methods:**

The study included 231 urban-dwelling asymptomatic 40–49 year old women. Body mass index (BMI), body fat percentage (BF%), blood glucose, blood lipids, blood pressure, and pulse wave velocity (PWV) were measured at rest. Cycle ergometer exercise tests were conducted to assess CRF as indicated by maximal oxygen uptake (VO_2max_). Participants were categorized into three CRF levels (low, moderate and high).

**Results:**

High CRF level was associated with significantly less BF%, lower PWV, and higher weekly physical activity compared with low and moderate CRF (*P <* 0.05). Compared to high CRF, the odds ratios for having ≥3 main CVD risk factors (overweight, hypertension, and dyslipidemia) in low and moderate CRF were 2.09 (95% CI: 1.48-2.94) and 1.84 (95% CI: 1.29-2.62), respectively. The proportion of participants with clinical ST segment depression and prolonged QTC interval during cycle ergometer testing was significantly higher in women with low CRF.

**Conclusions:**

Overall, Chinese middle-aged women demonstrated a moderate level of CRF. CRF was independently associated with CVD risk factors, including overweight, hypertension, dyslipidemia, arterial stiffness, and abnormal ECG during exercise, with the least fit women exhibiting the highest number of CVD risk factors.

## Background

With China’s rapid economic development, cardiovascular disease (CVD) has become the leading cause of death among Chinese adults [1]. Among Chinese women, the five leading causes of death are diseases of the heart, cerebrovascular disease, malignant neoplasms, pneumonia/influenza, and infectious diseases [1]. Physical inactivity and CVD risk factors such as obesity, hypertension, and dyslipidemia, are significantly related to the all-cause mortality in China [1]. Research suggests control of CVD risk factors and increased physical activity (PA) are two important strategies for reducing the burden of premature death among Chinese adults [1-3]. There is strong and consistent evidence indicating PA and cardiorespiratory fitness (CRF) independently predict the risk of CVD [4]. As an objective measurement of PA, CRF plays an important role in the relationship between PA and CVD, and could avoid the limitation of some studies in which PA was self-reported and participative. Recent studies [5,6] have demonstrated higher levels of CRF are associated with a favorable CVD risk profile, while women with lower CRF have an increased prevalence of CVD. Thus, effective improvement in PA and CRF may have significant implications for the clinical and public health burden of CVD [7-10].

To our knowledge, although a small number of previous studies have explored the relationship between self-reported PA and CVD [3,11], the association between CRF and CVD risk factors in Chinese middle-aged women has not been reported. Also, the relationship of CRF with fatness, arterial stiffness and abnormal ECG during exercise testing is still unclear. Therefore, the purpose of this study was to investigate the relationship between objectively measured CRF and the CVD risk factor profile, including overweight, hypertension, dyslipidemia, arterial stiffness, and abnormal ECG during exercise, in Chinese middle-age women.

## Methods

### Participants

The current study was conducted at Beijing Sport University from May to December in 2010. Four hundred and sixty-nine women 40–49 years of age living in the urban area of Beijing participated in a larger CVD surveillance study and completed an extensive questionnaire. Personal information included social background (age, sex, family income, and education), smoking, alcohol consumption, food frequency, previous disease history and family history. PA was assessed with the long Chinese version of the International Physical Activity Questionnaire (IPAQ) [12]. The IPAQ assesses PA undertaken for seven consecutive days across a comprehensive set of domains including leisure time PA, household and gardening (yard) activities, work-related PA, and transport-related PA. Computation of the total score requires summation of the duration (in minutes) and frequency (days) for all the types of activities in all domains. The final unit of PA is expressed as MET · minutes · week^−1^.

Two hundred and thirty-one women (age: 44.5 ± 3.3 yr, BMI: 23.8 ± 3.5 kg · m^−2^) voluntarily agreed to undertake additional laboratory tests. Test contents and requirements were confirmed with all participants by telephone two days prior to visiting the lab. Each participant was free of any exercise testing contraindications [13]. The study was approved by the Ethical Committee of Beijing Sport University. The nature, benefits, and risks of the study were explained to prospective participants and written informed consent was obtained before involvement in the study.

### CVD risk factors measurement

Height and weight were measured by standardized procedures [13]. Body mass index (BMI) was calculated as weight divided by height squared (kg · m^−2^). Waist circumferences (WC) were assessed at the thinnest position between the lowest rib and iliac crest [13]. Standard bioelectrical impedance analysis (BIA) assessments (Biospace Inbody 3.0) were performed with electrodes placed at the upper and lower extremities to determine body fat percentage (BF%).

Systolic blood pressure (SBP) and diastolic blood pressure (DBP) were measured with a Digital Blood Pressure Monitor (BIOspaceTM-2655/P) with participants in the upright-seated position. Participants rested for at least 5 minutes before the measurements. A minimum of 2 readings were obtained at intervals of at least 1 minute, with the average of those used to represent the participants’ blood pressure. If there was more than 5 mm Hg difference between the first and second reading, additional (1 or 2) readings were obtained, with the average of these multiple readings used.

Biochemistry measurements were assessed at 8:00 am on the test day following a 12-hour fast. Fasting blood glucose (FBG) was assessed by Roche Blood Glucose Meter (ACCU-CHEK Active). Triglyceride (TG) and total cholesterol (TC) were assessed by endpoint method [13]. High-density lipoprotein cholesterol (HDL-C) and low-density lipoprotein cholesterol (LDL-C) were measured by direct testing method [13]. For tests of TG, TC, HDL-C and LDL-C, kit products manufactured by Beijing Lead-man Biochemistry Co., Ltd. were used, with a Hitachi 7020 automatic biochemical analyzer utilized to attain final data for analysis.

Brachial-ankle pulse wave velocity (PWV) and Ankle Brachial Index (ABI) were measured in a quiet temperature-controlled room. A volume-plethysmograph(OMRON BP-203RPE) was employed to record PWV, ABI, BP, electrocardiogram (ECG), and heart sounds simultaneously. Participants were examined in the supine position, with ECG electrodes on both wrists, a microphone for detecting heart sounds on the left edge of the sternum, and cuffs wrapped on both the brachia and ankles. Cuffs were connected to a plethysmographic sensor to determine volume pulse form and an oscillometric pressure sensor to measure BP. Pulse wave for the brachium and ankle were stored, and sampling time was 10 seconds with automatic gain analysis and quality adjustment. After instrumentation, participants rested in the supine position for at least 10 minutes before data collection.

We used CVD risk factor thresholds according to American College of Sport Medicine (ACSM) to evaluate the CVD risk [13].

### Exercise test

During interviews, we learned that most participants had no or few experiences of exercise training on treadmills. However, they were very familiar with bicycle riding with a large majority of the women using a bicycle for daily commuting. Therefore, we conducted incremental cycle ergometer tests on an electronically braked ergometer (Merits Custo Med EC3000) to measure CRF. Participants were instructed abstain from any strenuous exercise on the day before testing. Maximal oxygen uptake (VO_2max_) was measured directly by collecting breathing gas with a mask. A gas analyzer (Cortex Metalyzer), which was validated in a previous study [14], recorded and computed the gas exchange. The analyzer was calibrated before each test. The initial workload was 0 W for one minute and increased 25 W every 2 minutes until the participant was exhausted or not able to maintain the required pedaling rate of 60 rpm [7]. During the test, ECG (Custo Med 12 guide Holter monitor) and BP (Suntec Tango^+^ monitor) were recorded. ST segment changes and corrected QT (QTC) interval were also recorded. The criteria for myocardial ischemia in ECG was horizontal or downsloping ST segment depression ≥ 1.0 mm at 80 msec after J point [13]. Abnormal QTC interval was considered as an affiliated measure of myocardial ischemia in addition to ST segment depression. The criteria for abnormal QTC interval was QTC interval <470 msec [13]. Rated Perceived Exertion (RPE) (Scale: 6–20) was used to test subjective feelings of fatigue and recorded 10 seconds before the end of each workload. The test was terminated when the participant reached peak VO_2max_[13] or showed any symptoms contraindicating continuation of exercise according to ACSM guidelines [13]. Based on previous researches [4,15], participants were categorized into three CRF groups (low, moderate and high). The least fit 20% were classified as low fit, the next higher 40% as moderately fit, and the top 40% as high fit.

### Data analysis

Differences in CVD risk factors related to overweight, hypertension, and dyslipidemia among the three CRF levels were examined by one-way ANOVA. Tukey post-hoc tests were used to test for differences between groups. Differences in proportions of participants with CVD risk factors were examined by chi-square analysis. Logistic regression tested the association between CRF levels and the number of CVD risk factors (overweight, hypertension, and dyslipidemia) with adjustment for age, family history, income, education, smoking, diet, alcohol consumption and total weekly PA. Differences of odds ratios of ST segment depression and long QTC interval during the cycle exercise test were also examined by logistic regression after adjustment of the covariates mentioned above and all other CVD risk factors. Repeated measure analysis was used to examine the trend of change in the ST segment and QTC interval during cycle exercise test. Data were analyzed with SPSS for Windows (version 21.0). *P <* 0.05 denoted statistical significance.

## Results

Demographic characteristics and CVD risk factors are displayed in Tables 1 and 2. The mean VO_2max_ was 27.35 ± 5.53 ml · kg^−1^ min^−1^, which was at a moderate level for the population’s gender and age [13]. The average total weekly PA was 3476.29 ± 3771.36 MET · min · week^−1^, which was at the high level of PA according to the IPAQ scoring protocol (high level: total PA >3000 MET-minutes/week). A majority (72.3%) of total weekly PA was comprised of non-leisure-time PA, including work-related PA (23.6%), transport-related PA (29.7%), and household-related PA (19.0%).

**Table 1:** Characteristics of participants

**Variable**	**Fitness level**^**a**^							
	**Low (N = 47)**		**Moderate (N = 92)**		**High (N = 92)**		**Total (N = 231)**	
	**Mean**	**SD**	**Mean**	**SD**	**Mean**	**SD**	**Mean**	**SD**
**Age (year)**	44.05	3.27	44.93	3.28	45.08	3.09	44.53	3.27
**Body Mass Index (kg · m**^**−2**^**)**	24.24	3.60	23.62	3.54	22.90	2.64	23.81	3.48
**Waist Circumference (cm)**	82.69	9.71	81.05	9.45	80.49	8.04	81.75	9.40
**Body Fat (%)**	32.07	6.11	28.75	5.25^**b**^	28.05	5.62^**d**^	30.21	5.96
**Fasting Glucose (mmol · L**^**−1**^**)**	5.44	1.13	5.52	0.85	5.56	1.62	5.49	1.10
**Total Cholesterol (mg · dl**^**−1**^**)**	168.84	137.98	139.59	127.26	149.95	87.21	157.40	130.07
**HDL-Cholesterol (mg · dl**^**−1**^**)**	52.78	10.30	57.90	12.97	50.53	9.90	54.29	11.40
**LDL-Cholesterol (mg · dl**^**−1**^**)**	127.33	116.94	97.55	44.01	113.60	27.10	116.17	93.22
**Systolic BP**^**e **^**(mmHg)**	115.01	13.47	114.82	14.81	117.41	14.47	115.25	14.07
**Diastolic BP**^**e **^**(mmHg)**	74.00	9.54	74.45	9.70	75.82	12.61	74.41	10.00
**Total PA**^**e **^**(MET · min · week**^**−1**^**)**	3058.6	2782.8	3368.7	3290.9	5273.9	6737.7^**d**^	3476.2	3771.3
**Total sitting time (min · week**^**−1**^**)**	1824.8	959.8	2020.5	990.6	2003.7	1077.5	1925.8	987.1
**Pulse Wave Velocity (cm · s**^**−1**^**)**	1345.21	182.70	1272.92	158.44	1220.64	168.36^**d**^	1262.17	169.74
**Ankle Brachial Index**	1.13	0.08	1.15	0.10	1.15	0.09	1.14	0.09
**VO**_**2MAX**_^**e **^**(mL · min**^**−1**^ **· kg**^**−1**^**)**	22.95	3.28	29.56	1.43^**b,c**^	36.37	4.60^**d**^	27.35	5.53

^**a**^According to Vo_2max_, the least fit 20% were classified as low fit, the next 40% as moderately fit, and the top 40% as high fit.

^**b**^Denotes significant difference (*P <* 0.05) between moderate and low CRF groups.

^**c**^Denotes significant differences (*P <* 0.05) between moderate and high CRF groups.

^**d**^Denotes significant difference (*P <* 0.05) between high and low CRF groups.

^**e**^BP refers to blood pressure, PA refers to physical activity, Vo_2max_ refers to maximal oxygen uptake during exercise testing.

**Table 2:** Proportion of cardiovascular disease risk factors in low, moderate and high cardiorespiratory fitness groups

**Variable**^**a**^	**Fitness level**^**b**^							
	**Low (N = 47)**		**Moderate (N = 92)**		**High (N = 92)**		**Total (N = 231)**	
	**N (%)**^**c**^		**N (%)**		**N (%)**		**N (%)**	
**Smoking**	2	(4.3%)	4	(4.3%)	6	(6.5%)	12	(5.2%)
**Alcohol drinking**	5	(10.6%)	20	(21.4%)	12	(12.5%)	37	(16.0%)
**Overweight by body mass index**	17	(36.5%)	27	(29.6%)	23	(25.0%)	67	(29.2%)
**Overweight by waist circumference**	12	(25.9%)	25	(26.8%)	15	(16.7%)	52	(22.6%)
**Overweight by body fat %**	30	(63.5%)	34	(36.6%)	31	(33.3%)	95	(41.1%)^**d**^
**Physical inactivity**	4	(8.2%)	5	(5.6%)	4	(4.2%)	13	(5.6%)
**High fasting glucose**	3	(5.9%)	9	(9.6%)	10	(11.1%)	22	(9.5%)
**High Total cholesterol**	9	(18.4%)	20	(21.4%)	23	(25.0%)	52	(22.5%)
**Low HDL- cholesterol**	4	(7.9%)	10	(10.7%)	8	(8.3%)	22	(9.5%)
**High LDL- cholesterol**	11	(23.7%)	20	(21.4%)	23	(25.0%)	54	(23.3%)
**High systolic blood pressure**	2	(5.3%)	9	(10.0%)	4	(4.5%)	15	(6.5%)
**High diastolic blood pressure**	4	(8.9%)	9	(10.0%)	4	(4.3%)	17	(7.5%)
**ST-Depression during exercise**	3	(6.4%)	5	(5.4%)	3	(3.3%)	11	(4.8%)^**d**^
**Long QTC during exercise**	3	(6.4%)	4	(4.3%)	3	(3.3%)	10	(4.3%)^**d**^

^**a**^Reference value for abnormalities according to American College of Sports Medicine criteria [13].

^**b**^According to VO_2max_, the least fit 20% were classified as low fit, the next 40% as moderately fit, and the top 40% as high fit.

^**c**^N denotes the number of participants with the CVD risk factor within each CRF group, % indicates the percentage of participants with the CVD risk factor within each CRF group.

^**d**^Denotes significant difference (*P <* 0.01) in percentages among the three CRF groups.

Overall, CVD risk profiles of the moderate and high CRF groups were more favorable than that of the low CRF group. There were significant differences in total weekly PA among the three CRF groups (*P <* 0.05), with total weekly PA being highest in the high CRF group and lowest in the low CRF group. Total weekly PA was significantly higher in the high CRF group than in the low CRF group (*P <* 0.05), with no significant difference between the moderate and low CRF group (*P >* 0.05). Higher CRF levels were also associated with significantly less BF% (*P <* 0.05) and lower PWV (*P <* 0.05), which indicated less stiffness of the vasculature. There were significant differences in BF% between the moderate and low CRF group (*P <* 0.05) and the high and low CRF group (*P <* 0.05). PWV was significantly lower in the high CRF group than in the low CRF group (*P <* 0.05). No significances were found in other variables (*P >* 0.05).

The chi-square test revealed significant differences in the proportions of abnormal BF%, ST segment depression, and long QTC interval across CRF groups (*P <* 0.05) (Table 2). The proportion of participants with overweight status, ST segment depression, and long QTC interval during cycle exercise testing were lowest in the high CRF group and highest in the low CRF group. No significances existed for those variables across CRF groups (*P >* 0.05).

After adjustment for a range of potential confounding factors including age, family history, income, education, smoking, diet, alcohol consumption and total PA, logistic regression indicated the odds ratio for having ≤1, 2, or ≥ 3 CVD risk factors (Table 3). Odds ratios for having ≥3 CVD risk factors in the low and moderate CRF groups were significantly higher compared with the high CRF group (*P <* 0.05). The likelihood of having ≥3 CVD risk factors in the low CRF group was 2.09 times greater than women in the high CRF group. Also, the low CRF group had significant less probability of having ≤1 CVD risk factors (*P <* 0.05). Table 3 also shows the that odds ratios of ST segment depression and long QTC interval during cycle exercise testing in the low and moderate CRF groups were significantly higher compared with the high CRF group (*P <* 0.05). This indicated the probability of myocardial ischemia in the high CRF group was significantly less than in the moderate and low CRF groups.

**Table 3:** Odds ratio of number (N) of cardiovascular disease (CVD) risk factors and abnormal electrocardiogram (ECG) according to cardiorespiratory fitness level

**Variable**^**a**^	**Fitness level**^**b**^						
	**Low**			**Moderate**			**High**^**e**^
	**OR (95% C.I.)**		**P-value**	**OR (95% C.I.)**		**P-value**	**(Reference)**
**N of CVD risk factors**^**c**^							
≥3 Risk factors	2.09	(1.48-2.94)	<0.01	1.84	(1.29-2.62)	<0.01	1.00
2 Risk factors	1.60	(1.13-2.26)	<0.01	1.10	(0.76-1.60)	0.61	1.00
≤1 Risk factors	0.71	(0.51-0.99)	<0.05	0.79	(0.58-1.10)	0.16	1.00
**Abnormality in ECG**^**d**^							
ST-Depression	2.14	(1.52-3.00)	<0.01	1.63	(1.14-2.32)	<0.01	1.00
Long QTC	2.06	(1.44-2.96)	<0.01	1.52	(1.04-2.23)	<0.05	1.00

^a^Reference value for abnormalities according to ACSM instructions [13].

^**b**^According to VO_2max_, the least fit 20% were classified as low fit, the next 40% as moderately fit, and the top 40% as high fit.

^**c**^Odds ratio (95% confidence interval) of number of CVD risk factors related to overweight, hypertension, and dyslipidemia adjusted for age, family history, income, education, smoking, diet, alcohol consumption and total weekly physical activity.

^**d**^Odds ratios of ST depression and long QTC (corrected QT) interval during the cycle exercise testing adjusted for age, family history, income, education, smoking, diet, alcohol consumption, total physical activity, and other CVD risk factors.

^e^High CRF was the referent for both models.

**Figure 1:**
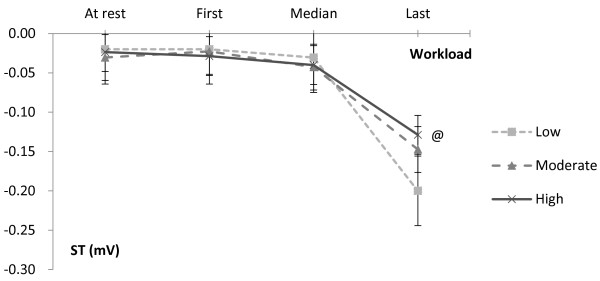
**ST Segment Change during Exercise Test.** The average values were recorded at rest, the first workload, the median workload and the last workload phase. @ denotes significant difference (*P <* 0.05) in the trend of change among the three CRF groups.

**Figure 2:**
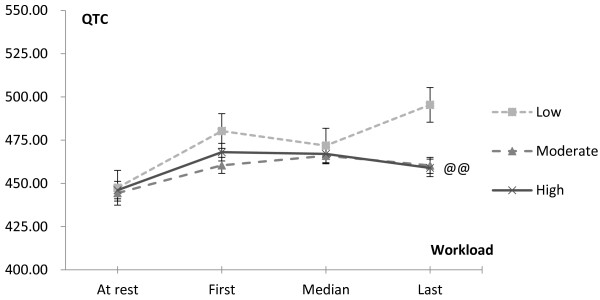
**QTC interval Change during Exercise Test.** The average values were recorded at rest, the first workload, the median workload and the last workload phase. @@ denotes significant difference (*P <* 0.01) in the trend of change among the three CRF groups.

Figure 1 shows the trend of ST segment changes during exercise testing among different CRF groups. There were significant differences between the low and moderate CRF group (*P <* 0.05) and the low and high CRF group (*P <* 0.05). There were no significant differences between the high and moderate CRF group (*P >* 0.05). These data indicated the potential of myocardial ischemia in both the high and moderate CRF groups was less than in the low CRF group. Figure 2 shows the pattern in QTC interval with significant differences between the low and moderate CRF group (*P <* 0.05) and the low and high CRF group(*P <* 0.05). There were no significant differences between the high and moderate CRF group (*P >* 0.05).

## Discussion

Beijing is the Capital of China with a population of approximate 16.85 million living in 11 urban districts. The burden of medical care for this large population has increased tremendously in recent years, especially for CVD [16,17]. The purpose of the current study was to examine the association between objectively measured CRF and several CVD risk factors in middle-aged women living in urban Beijing. To the best of our knowledge, this is the first study to explore this topic in a Chinese female population. The results of the present study demonstrated that CRF was significantly inversely associated with CVD risk in this population with both moderate and high levels of CRF being associated with reduced risk.

CRF is primarily a function of the heart’s maximal ability to pump blood and the ability of skeletal muscle to extract and use oxygen. It is a more objective measure reflecting the PA level and cardiovascular function than self-reported PA which is prone to bias [15]. CRF is influenced by both genetic and environmental factors. Environment, such as the frequency, intensity and duration of daily PA, has a significant effect on CRF, and appears to account for more of the variation in CRF than do genetic factors.

We observed that CRF was strongly and inversely associated with BF%. As a more precise variable, BF% measured by BIA indicated an association between fatness and CRF, while BMI failed to exclude the confounding effect of lean body mass. Regular PA has been shown to reduce the risks for obesity and metabolic disease at all ages. Recent studies [4,18-20] support the finding that higher CRF is related to lower risks of obesity for both men and women. For example, Irwin et al. [21] reported higher levels CRF predicted less body weight and lower BF%. One study conducted in Hong Kong [22] also showed a significant inverse association between BF% and CRF in women aged 55–94 years. Similar results from Wong et al. [23] demonstrated body fat mass and lean body mass were independently associated with VO_2max_ in Chinese adults. The CRF level measured in our study was also consistent with results for adult women aged 40–49 years old who performed cycle exercise tests in these two studies [22,23].

CRF was also significantly negatively associated with PWV, which is a valid and reliable measurement of arterial stiffness. PWV indicates the general burden of atherosclerotic disease and subclinical damage from multiple risk factors over time, such as the effects of aging, smoking, and lipid metabolism [24]. In alignment with our results, recent studies have also discovered that CRF is associated with arterial stiffness. Boreham et al. [25] reported CRF was inversely related to PWV in young adults, and the relationship was independent of other CVD risk factors and daily PA levels. Gando et al. [26] reported higher CRF was associated with lower BP and arterial stiffness in older women. One possible reason for the relationship is that higher CRF minimizes age-related structural changes in the arterial wall related to an elevated overall content of elastin and a reduced calcium content [27]. Another possibility is higher CRF may act to maintain endothelium-dependent vasodilation [28].

We did not observe significant differences in FBG, TC, HDL-C, LDL-C, SBP, and DBP among the three CRF groups. This may be partially due to the inclusion of participants asymptomatic of CVD. Also, participants might suffer from diverse CVD risk factors which made it harder to detect a difference in the general CVD risk profile across participants. Thus, we used ACSM’s risk factor stratification [13] to summarize and evaluate the CVD risk within the sample. We used overweight, hypertension, and dyslipidemia as the main CVD risk factors as previous research and theories [5,29,30] support the relationship between these factors and CRF. We also controlled for age, family history, smoking, total weekly PA and other factors to clarify the relationship. The results of revealed women with high to moderate CRF were significantly less likely to have ≥3 CVD risk factors, and more likely to have ≤1 CVD risk factors. Therefore, CRF was inversely associated with the risk of CVD.

Furthermore, the results (Table 3, Figures 1 and 2) from the ECG during exercise testing disclosing greater myocardial ischemia in women with the lowest CRF also supports the association mentioned above. Some CVD risk factors might fail to provide prognostic information of CVD among an asymptomatic population, while results from exercise testing can help guide decisions regarding CVD risk evaluation. It is suggested that exercise testing be recommended for adults with the presence of at least one conventional CVD risk factor [13], although the prognostic importance of exercise-induced ST segment depression and long QTC interval still needs clarification. Nonetheless, similar to our findings, other studies have also observed an association between CRF and abnormal ECG during exercise testing. Lakka et al. [31] reported higher levels of both leisure-time PA and CRF had a strong, graded, inverse association with the risk of ST segment depression during exercise and acute myocardial infarction during follow-up. Church et al. [32] also noticed a significant relationship among CRF, abnormal exercise ECG, and future CVD mortality. Our study included ECG during exercise testing as an important aspect in the evaluation of CVD risk. This is the first report of the significant relationship between CRF and odds ratios of ST segment depression and long QTC interval in any Chinese female population.

The results regarding the relation between CRF and CVD risk factors were in agreement with the conclusions of a series of reports with non-Chinese women [5,18,30,33,34]. A cross-sectional study from Kokkinos and colleagues [30] showed women in the lowest CRF category had a less favorable CVD risk profile than those in moderate and high CRF categories. Haddock et al. [5] reported higher CRF levels were significantly associated with lower TC, TG, and higher HDL in women regardless of their hormonal status. Longitudinal studies have also confirmed these results. Blair et al. [15] reported moderate CRF had a protective effect against CVD risk and reduced the risk of premature mortality. Kodama et al. [10] indicated increased CRF was associated with lower risk of all-cause mortality and incidence of CVD. However, most studies have been based on white and high-income populations in western countries, mainly the United States. To date, there is no similar report focused on a Chinese population.

Interestingly, our results also indicated total weekly PA was much higher than the average level demonstrated by women in western developed countries (i.e., more than five times the recommended dose in the American PA Guidelines) [33]. Our PA findings approximate data (4672 ± 1692 MET · min · week^−1^) also collected by IPAQ in the Guangzhou Biobank Cohort Study [35]. Additionally, the time spent in leisure-time PA among the current study’s middle-aged women was relatively low (27.7%) while the transport-related PA was relatively high (29.7%) compared to that observed in western developed countries [33]. The proportion of time spent in these two types of activities is similar to a previous study focused on commuting, leisure-time PA, and CVD risk factors in China [3]. The reason for the higher levels of transport-related PA is that China is the largest bicycle-using country in the world, and most Chinese living in urban settings use a bicycle, utilize public transportation, or walk to and from work, shopping or school [35]. Also, the retirement age for Chinese women is between 50–55 years old, depending on the type of job. Most of our participants were full-time workers with additional responsibilities to take care of family members at home. Therefore, their total weekly PA is high. Although leisure-time PA has been thought to be more efficient in improving health-related factors, recent research [11,33] has shown total PA is the key variable associated with a more favorable CVD risk profile.

This study is the first to document the total PA by IPAQ and directly-measured CRF levels in Chinese middle-aged women. It is also the first study to report the association between CRF and CVD risk in this unique female population. Moreover, we have documented an inverse association between CRF and fatness, and also added various CVD risk factors in the evaluation, such as PWV at rest and ECG during exercise testing, providing a more comprehensive picture of the CVD risk profile.

The current study also had some limitations. First, it was a cross-sectional study, and it does not disclose a causal relationship between CRF and CVD risk. Second, the sample was restricted to one age group and geographic area. Further studies are needed to generalize the conclusions to a larger population with different ages and geographic regions. Also, despite the common use of bicycles among these women, the cycle exercise test might underscore the CRF of these women by 5-10% compared with a treadmill test. Nonetheless, our results add value to the existing data highlighting the association between CRF and CVD risk profile with implications toward the prevention of CVD in Chinese women.

## Conclusions

The total weekly PA of this population was high, and mainly consisted of non-leisure-time PA. Moderate and high levels of CRF measured by a cycle exercise test were significantly related to the CVD risk profile in 40–49 year old Chinese women living in the urban district of Beijing. It seems reasonable to suggest a stronger emphasis be placed on increasing the CRF level of this population via regular PA, especially for those who are unfit. Further studies need to confirm the causal relationship between CRF and CVD risk for Chinese and Asian female populations.

## Competing interests

The authors declare that they have no competing financial interests exist.

## Authors’ contributions

WZ, JC and SH conceived the study, completed most of the statistical analyses, and drafted the manuscript. MX and HS carried out the design and implementation of the study. YS helped to interpret the data and draft the manuscript. All authors have read and approved the final manuscript.

## Pre-publication history

The pre-publication history for this paper can be accessed here:

http://www.biomedcentral.com/1472-6874/14/62/prepub
